# Draft Genome Sequence of Burkholderia gladioli Strain UCD-UG_CHAPALOTE (Phylum *Proteobacteria*)

**DOI:** 10.1128/genomeA.01462-14

**Published:** 2015-01-22

**Authors:** Cassandra L. Ettinger, Hanan R. Shehata, David Johnston-Monje, Manish N. Raizada, Jonathan A. Eisen

**Affiliations:** a Genome Center, University of California Davis, Davis, California, USA; b Department of Plant Agriculture, University of Guelph, Guelph, Ontario, Canada; c Department of Microbiology, School of Pharmacy, Mansoura University, Mansoura, Egypt; d Department of Evolution and Ecology, University of California Davis, Davis, California, USA; e Department of Medical Microbiology and Immunology, University of California Davis, Davis, California, USA

## Abstract

Here, we present the draft genome of Burkholderia gladioli strain UCD-UG_CHAPALOTE. This strain is an endophyte isolated from surface sterilized seeds of an ancient Mexican landrace of corn, Chapalote. The genome contains 8,527,129 bp in 109 scaffolds.

## GENOME ANNOUNCEMENT

Members of Burkholderia gladioli (formerly Pseudomonas marginata) are aerobic Gram-negative rod-shaped soil bacteria (1). Representatives of this species include plant pathogens, opportunistic human pathogens, and endophytes (2–4). B. gladioli strain UCD-UG_CHAPALOTE is an endophyte that was isolated from surface sterilized seeds of an ancient landrace of Mexican corn (Zea mays subsp. *mays*) known as Chapalote originating from the State of Sinaloa (5). The endophyte was isolated at the University of Guelph, Guelph, Ontario, Canada during August 2008 as part of an ongoing project investigating the antifungal properties of endophytic bacteria (6). Genomic DNA was extracted at the University of Guelph using a bacterial genomic DNA isolation kit (catalog no. 17900; Norgen Biotek Corp) according to the manufacturer’s recommendations and DNA was ethanol precipitated before being shipped to the University of California Davis for library preparation, sequencing, and analysis. Illumina paired-end libraries were made using a Nextera DNA sample preparation kit (Illumina) and libraries were sequenced on an Illumina MiSeq with a read length of 250 bp.

A total of 3,858,496 paired-end reads were produced, and after quality trimming and error correction, 3,729,095 high-quality reads were retained. Sequence processing and assembly were performed using the A5 assembly pipeline (version A5-miseq 20140604) following the workflow described by Dunitz et al. (7, 8). The A5 assembly pipeline automates the processes of data cleaning, error correction, contig assembly, scaffolding, and quality control. The assembly resulted in 150 contigs that were contained in 109 scaffolds (minimum, 508 bp; maximum, 471,925 bp; *N*_50_, 148,354 bp). The final assembly contained 8,527,129 bp with a GC content of 67.75% and a median coverage of 98×. Genome completeness was assessed using Phylosift software (version 1.0.1), which searches for 37 highly conserved, single copy marker genes (9, 10). All of the marker genes were found in the final assembly.

Automated annotation was performed using the RAST server (11). B. gladioli strain UCD-UG_CHAPALOTE contains 7,721 predicted protein coding sequences and 74 predicted non-coding RNAs. Previous sequencing of the 16S rRNA gene identified this isolate as a member of B. gladioli. A full-length (1,490-bp) 16S rRNA gene sequence was obtained from the RAST annotation and was used to verify the identity of the Burkholderia species by aligning it to 16S rRNA gene sequences from 59 Burkholderia isolates and an archaea outgroup from the Ribosomal Database Project (RDP) (12). This alignment was used to construct a phylogenetic tree using FastTree 2 (http://dx.doi.org/10.6084/m9.figshare.1245105) (13). Additionally, the full-length 16S sequence was found to be 100% identical to publically available B. gladioli 16S sequences confirming the species identity of this isolate.

### Nucleotide sequence accession numbers.

This whole-genome shotgun project has been deposited at DDBJ/EMBL/GenBank under the accession no. JRGO00000000. The version described in this paper is version JRGO01000000. The raw Illumina reads are available at ENA/SRA accession no. PRJEB7719 (ERP008658).
